# Symptom Clusters, Psychological Distress, and Quality of Life in Patients with Atrial Fibrillation

**DOI:** 10.3390/healthcare11091353

**Published:** 2023-05-08

**Authors:** Chohee Bang, Sookyung Park

**Affiliations:** 1 Department of Nursing, College of Health Science, Honam University, Gwangju 62399, Republic of Korea; choice3bch@naver.com; 2 School of Nursing, Korea University, Seoul 02841, Republic of Korea

**Keywords:** atrial fibrillation, symptom cluster, psychological distress, quality of life, depression, anxiety, nurses/midwives/nursing

## Abstract

**Background:** Patients with atrial fibrillation (AF) experience diverse symptoms such as palpitations, dizziness, and fainting that lead to depression, anxiety, and poor quality of life. Management of symptoms is fundamental for AF, and with the increasing prevalence of AF, studies on management of symptoms in patients with AF are needed. **Objectives:** This study aimed to assess symptom clusters according to symptom severity in patients with atrial fibrillation and evaluate the relationships between symptom cluster groups and the psychological distress and quality of life of these patients. **Design:** A descriptive survey was used in this study. **Methods:** A total of 175 patients were included in this study. Data regarding symptoms, psychological distress, and quality of life were obtained using structured questionnaires and analyzed using frequency and percentage, mean and standard deviation, cluster analysis, *t*-testing, Chi-square testing, Pearson’s correlation coefficient, and multiple regression analysis. The Euclidean distance square of the hierarchical cluster was used to form symptom cluster groups. **Results:** Two groups of symptom clusters were formed based on the seven most common symptoms (i.e., chest palpitations, fatigue/tiredness, dizziness, lack of energy, pulse skipping, insomnia, and heavy breathing) of atrial fibrillation patients. Psychological distress and quality of life showed significant correlations with the symptom cluster groups (*p* < 0.001). **Conclusion:** Symptoms of atrial fibrillation increased patients’ depression and anxiety, and further affected their quality of life. Therefore, management of symptoms is critical to maintaining a high quality of life. Nursing interventions based on the characteristics of symptom cluster groups must be developed and attempted.

## 1. Introduction

Atrial fibrillation (AF) is a common arrhythmia in clinical settings. AF causes the atrium to beat in an irregular pattern, resulting in irregular and fast waveforms and an irregular contraction of the ventricle [1]. The prevalence of AF is rising globally. In South Korea, it has more than doubled, from 0.73% in 2006 to 1.53% in 2015, and is expected to rise to 5.6% by 2060 [2]. AF symptoms vary. They include not only physical symptoms and discomfort, such as shortness of breath, dizziness, chest pain, and fainting, but also psychological symptoms such as frustration, anxiety, depression, lethargy, and insomnia. Although the mechanism is unknown, some patients exhibit no symptoms [3,4]. Patients often believe that their physical discomfort is the result of the heart, which is regarded as the most important organ in the body. These thoughts heighten mental anxiety, leading to insomnia and depression [5]. These symptoms cause psychological distress and have a significant impact on the quality of life of patients with AF [6].

Most chronic disease patients have multiple symptoms simultaneously. These concurrent symptoms are referred to as symptom clusters. Understanding the relationships between multiple symptoms in the same symptom cluster can aid in symptom control and management [7]. Because uncontrolled symptoms are the leading cause of poor quality of life, identifying symptom clusters and seeking appropriate interventions are critical steps in managing patients’ complex symptoms and improving their quality of life [8]. Because AF patients have a wide range of symptoms, symptom management is critical. Therefore, the goals of this study were to identify AF symptom clusters by assessing patients’ experiences with various symptoms and to evaluate whether subgroups of AF patients could be identified based on the most common symptoms they experienced. Furthermore, the associations between symptom cluster groups, psychological distress, and quality of life were investigated.

## 2. Background

As the symptom management concept has grown in importance in nursing, it has been proposed that nursing interventions for groups of similar and related symptoms are more effective than the treatment of individual symptoms [9]. Symptom clusters are groups of two to three related symptoms that are independent of one another. Clusters serve as the foundation for identifying and diagnosing conditions related to the disease or its treatment. They also help to understand the relationships between symptoms within clusters, which may lead to control of other significant symptoms [7,8,10]. There are two approaches to studying symptom clusters. The first method is to assess similar group symptoms. The second method is to choose a few symptoms and group patients together based on the severity and frequency of the symptoms [10]. The second method can reveal the characteristics of groups of patients who have multiple severe symptoms simultaneously. These discoveries can aid in the planning of effective nursing interventions to alleviate symptoms collectively rather than individually [10]. For patients with AF, severe symptoms include palpitations, dizziness, pre-syncope, and shortness of breath [4].

In this study, the second method of examining clusters was used to determine symptom clusters in patients with AF. In previous studies of various patient groups, symptom clusters have been shown to affect patients’ quality of life, psychological distress, and physical function [11,12]. Thus, a systematic evaluation of symptoms and a clear understanding of symptom clusters can help improve health-related outcomes by revealing the functional status of patients with AF. In a study that classified symptom clusters in patients with chronic diseases and cancer, the demographic and clinical characteristics of groups with severe symptoms were not different from patient groups with mild symptoms [13]. Other studies, however, have reported contradictory results. In a study that compared the severity of pain and fatigue in patients classified as having severe or mild symptoms, the group with severe symptoms was younger, mostly unmarried, or living alone [14]. Another study categorized the demographic and clinical characteristics of breast cancer patients undergoing postoperative chemotherapy and was grouped into clusters based on symptom severity. The proportion of patients with stage I breast cancer was higher than that of patients with stage II breast cancer, in a group of patients with severe symptoms [15]. Similarly, assessing the demographic and clinical characteristics of patients with AF may aid in the prediction of patients with severe symptoms and the provision of effective and proactive nursing interventions.

Psychological distress of patients with AF, including depression and anxiety, is linked to the frequency and intensity of symptoms [16]. Anxiety raises the frequency of symptoms, and the more frequent the symptom, the more severe the symptom experience. Therefore, the increased experience of symptoms contributes to increased anxiety and depression [17,18,19]. According to other studies, anxiety aggravates symptoms and causes psychological distress [6,20]. When patients with AF experience symptoms, they and their caregivers or supporters face physical and psychological stress, as well as problems in daily and social life, which leads to a lower quality of life [21]. Understanding symptom clusters can aid in the prediction and management of symptoms when they first appear, lowering symptom management costs by dealing with multiple symptoms earlier and more effectively [22]. Identifying symptom clusters that affect the quality of life can provide a scientific foundation for nursing interventions that improve patient’s quality of life [22]. This study aimed to identify AF patient symptom clusters, assess whether groups were formed based on symptom clusters, and investigate the relationships between symptom cluster groups, psychological distress, and quality of life.

## 3. Purposes of the Study

This study was conducted to assess symptoms of patients with AF, identify symptom clusters according to the severity of the symptoms, and evaluate the relationship of symptom cluster groups with psychological distress and quality of life.

## 4. Method

### 4.1. Design

A descriptive survey was employed to assess if symptom clusters were formed based on the frequency and intensity of various symptoms experienced by patients with AF, and to understand the relationships among cluster groups, psychological distress, and quality of life.

### 4.2. Sample and Setting

Adults over the age of 18 years who were diagnosed with AF by the Department of Cardiology at a single medical center in South Korea, who were undergoing treatment in an outpatient clinic, emergency room, or hospital and were able to communicate were eligible as study participants. Patients who were diagnosed with a severe mental illness and prescribed antidepressants, or who were diagnosed with diseases other than AF within the previous three months, were excluded from the study. The optimal number of participants for cluster analysis is 4–5 times the number of variables, and a minimum of 100 participants is recommended [23]. In this study, the 16 symptom checklist (SCL) items were used for the cluster analysis. Thus, a total of 175 subjects, including 160 patients and an additional 15 subjects for a 10% dropout rate, were selected. Data on clinical characteristics were obtained from medical records. All the questionnaires that were used were previously adapted to the Korean language and showed sufficient reliability and validity. They were tested in patients with AF in previous studies and demonstrated good reliability and validity in this study.

### 4.3. Procedures

#### 4.3.1. Symptom Clusters

Symptom clusters are two or more symptoms of a disease that appear simultaneously. The symptom checklist version III (SCL III) (created by Bubien et al. and modified by Jenkins) [24] was used to assess the symptoms of patients with AF; factor analysis was used to identify symptom clusters. The SCL comprises 16 items that assess AF symptoms. The frequency and severity of symptoms are assessed on a 5-point scale ranging from 0 (none) to 5 (high frequency), and on a 4-point scale from 0 (no experience) to 3 (very severe), respectively; the higher the score, the greater the frequency and severity of symptoms. In this study, Cronbach’s α values for item frequency and severity were 0.831 and 0.813, respectively.

#### 4.3.2. Psychological Distress

Psychological distress is defined as a state of anxiety, sadness, lethargy, rejection, and/or resentment caused by disease experiences [25]. Anxiety and depression were examined in this study.

Anxiety was assessed using the State-Trait Anxiety Inventory (STAI, 1971), which was developed by Spielberger and standardized in Korean. The tool comprises 20 items that are scored on a 4-point scale; the higher the score, the greater the anxiety. Cronbach’s α was 0.953 in this study.

Depression was assessed using the Center for Epidemiologic Studies–Depression (CES–D) scale, developed by Radloff [26], and standardized in Korean. The tool comprises 20 items evaluated on a 4-point scale; the higher the score, the greater the depression. Cronbach’s α was 0.948 in this study.

#### 4.3.3. Quality of Life

The 36-item Short Form Health Survey (SF-36) version II, which is widely available and standardized in Korea, was used to evaluate the quality of life. The tool consists of eight health domains and one health change domain, which were assessed using 36 items. The tool is scored summarily; the higher the score, the better the quality of life. Cronbach’s α value was 0.704–0.952 in this study.

#### 4.3.4. Demographic and Clinical Characteristics

The demographic characteristics examined in this study include gender, age, education level, occupation, monthly income, cohabitation with family, alcohol consumption, smoking, and drug use. Clinical characteristics included AF type, history of radiofrequency catheter ablation, disease duration, other chronic diseases, cardiac output coefficient, New York Heart Association (NYHA) Functional Classification, and number of hospital visits due to symptoms.

### 4.4. Ethical Considerations

This study was carried out following the principles outlined in the Declaration of Helsinki. Furthermore, this study was authorized by the ethics committee at the Institutional Review Board of the medical institution, where the principal investigator works. Participants completed the survey after being informed about the study’s purpose and process and signing an informed consent form. We explained that there would be no consequences if the participants dropped out of the study. All participants were compensated for their lost time.

### 4.5. Data Analysis

The collected data were assessed using SPSS WIN 23.0 (IBM Corp., Armonk, NY, USA). The subjects’ demographics, clinical characteristics, depression and anxiety levels, AF symptoms, and quality of life were analyzed using frequency, percentage, mean, and standard deviation (SD). SCL-ascertained symptom frequency and severity were analyzed using frequency, percentage, mean, and SD. Nine symptoms were excluded because 70% of the subjects did not experience them. The remaining seven significant symptoms were assessed via hierarchical clustering analysis using Ward’s method and cluster analysis using the square Euclidean distance scale, to identify two groups: one with a high frequency of symptoms and another with a low frequency of symptoms.

Among the many clustering analysis methods available, hierarchical clustering analysis was chosen and used because it creates large clusters from small clusters. Ward’s method was chosen for clustering because it identifies clusters based on the variance of measurement values for all subjects in a cluster. For each clustering step, the pair with the smallest variance in measurements between each subject was clustered. The Euclidean distance was used as a measure of dissimilarity. The number of clusters was determined by changes in the hierarchical clustering correlation index and the researcher’s discretion. Chi-square and Student’s *t*-tests were used to compare non-continuous and continuous variables of demographic and clinical characteristics of groups based on symptom clusters. The relationships between depression, anxiety, quality of life, and all variables were assessed using Pearson’s correlation coefficient. Variables with a *p*-value of ≤0.05 were chosen for regression analysis to assess factors that affected depression, anxiety, and quality of life.

## 5. Results

### 5.1. Demographic and Clinical Characteristics

The mean age of the subjects was 61.55 ± 11.50 years, and 74.9% of them were male. Of the participants, 81.7% lived with their families, and 78.9% said their families provided emotional support. Paroxysmal AF was more common (62.3%) than persistent AF, and the subjects had an average of 1.72 ± 1.85 comorbidities. Hypertension (44.6%) was the most widely reported comorbidity. Other comorbidities reported by participants included diabetes mellitus (21.7%), coronary artery disease (21.7%), heart failure (21.1%), and arrhythmias (not AF) (20.6%). During the previous six months, 62.3% of the subjects had scheduled, regular visits to the hospital. In contrast, 37.7% of the subjects visited the hospital because their symptoms had worsened. The average duration of the disease was 5.82 ± 5.061 years (Table 1).

### 5.2. Symptoms of AF Psychological Distress and Quality of Life

The frequency of AF symptoms was 7.29 ± 6.45 points. The most common symptoms were heart racing, fatigue, dizziness/light headache, tiredness/lack of energy, heart fluttering/skipping, difficulty sleeping, and shortness of breath. The severity of AF symptoms was calculated as 7.43 ± 5.05 points. The most severe symptoms were heart racing, fatigue, dizziness/light headache, tiredness/lack of energy, difficulty sleeping, and heart fluttering/skipping, shortness of breath (Table 2). Anxiety levels ranged from 24 to 64 points, with an average of 39.94 ± 9.94 points. Depression scores ranged from 1 to 42 points, with an average of 13.10 ± 8.88 points indicating mild depression overall. The overall physical health-related quality of life score was 49.99 ± 10.67 points. The subdomain of physical role limitation indicated the highest score, followed by physical function and pain. General health had the lowest score. The average mental health-related quality of life was 50.00 ± 8.88 points. Emotional role limitation had the highest score in this category, followed by average social function and mental health. The vitality level had the lowest score (Table 3).

### 5.3. Symptom Clusters

The frequency and severity of the SCL’s 16 symptoms were investigated. The nine symptoms that 70% of the subjects did not experience were exempted. Two symptom cluster groups (group 1, *n* = 121; group 2, *n* = 54) were created using the remaining seven significant symptoms (i.e., heart racing, fatigue, dizziness/light headache, tiredness/lack of energy, heart fluttering/skipping, difficulty sleeping, and shortness of breath). Cluster analysis of the seven symptoms in 175 subjects revealed that the frequencies of the seven symptoms differed significantly between the two groups (*p* < 0.001) (Table 4). Fewer subjects in cluster group 1 lived with their families and received family support when compared to subjects in cluster group 2. Cluster group 1 also had more comorbidities than cluster group 2. The frequency of arrhythmia-related procedures, hospital visits due to symptoms, and disease duration were also significantly different between the two groups (Table 5).

### 5.4. Relationship between Symptom Cluster Groups and Psychological Distress

A comparison of depression and anxiety levels between the two groups revealed statistically significant differences (*p* < 0.0001) (Table 5). The relationship between the depression and anxiety variables was assessed using Pearson’s correlation coefficient, to determine whether symptom cluster groups affected depression and anxiety. Concerning depression, variables with Pearson’s correlation coefficient *p*-values of ≤0.05 were smoking, cardiac output coefficient, NYHA class, number of visits to the hospital due to symptoms, and symptom cluster group variables. Regarding anxiety, variables with Pearson’s correlation coefficient *p*-value of ≤0.05 were alcohol consumption, cardiac output coefficient, NYHA class, number of visits to the hospital due to symptoms, number of comorbidities, and symptom cluster group variables. After excluding symptom cluster group variables as covariates, regression analysis revealed that symptom cluster group variables had significant effects on depression and anxiety (*p* < 0.0001) (Table 6).

### 5.5. Relationship between Symptom Cluster Groups and Quality of Life

Cluster group 1 had a low quality of life in terms of physical health, mental health, and all other domains; quality of life in terms of physical health was lower in group 1 than the quality of life in terms of mental health (*p* < 0.0001) (Table 5). The relationship between quality of life and other variables was assessed using Pearson’s correlation coefficient, to determine whether the cluster group affected patients’ quality of life. Variables with Pearson’s correlation coefficient *p*-values of < 0.05 between physical health-related quality of life and other variables were anxiety, depression, alcohol consumption, smoking, number of comorbidities, cardiac output coefficient, NYHA class, number of visits to the hospital due to symptoms, and symptom cluster group variables. Variables with a Pearson’s correlation coefficient *p*-value of ≤ 0.05 between mental health-related quality of life and other variables include anxiety, depression, alcohol consumption, smoking, arrhythmia, cohabitation with family, family support, religion, age, and symptom cluster group variables. Regression analysis was performed after excluding symptom cluster group variables as covariates, and it was discovered that these variables significantly impacted physical and mental health and quality of life. Therefore, cluster group 2, which had a lower frequency of symptoms than cluster group 1, had a higher quality of life in terms of physical and mental health (Table 6).

## 6. Discussion

This study evaluated the symptoms of patients with AF, symptom clusters, demographic and clinical characteristics of each symptom cluster group, and the connection between symptom cluster groups, psychological distress, and quality of life. From a total of 16 symptoms, the frequency and severity of seven prevalent and significant symptoms were used to create two symptom cluster groups. The variables that differed significantly between the two cluster groups were marital status, cohabitation with family, family support, number of comorbidities, cardiac output coefficient, NYHA class, history of arrhythmia surgery, number of hospital visits due to symptoms, and disease duration. The group with the highest frequency of symptoms had higher anxiety and depression, as well as a lower quality of life.

In this study, the frequency and severity of symptoms were lower than in previous studies [20,27]. This divergence could be attributed to the variety of AF symptoms, asymptomatic AF, and/or the possibility that experiencing multiple symptoms simultaneously could lead to a muddled expression of the symptoms [28]. Furthermore, a higher proportion of subjects in our study had paroxysmal AF, and many of the subjects were outpatients; both of these factors may have reduced the severity of the reported symptoms. AF is classified using the duration of arrhythmia episodes and the pattern of episode termination. The fact that paroxysmal AF resolves in seven days may have influenced the frequency and severity of symptoms in this study. The most common and severe symptoms in this study were heart racing, fatigue, dizziness/light headache, tiredness/lack of energy, heart fluttering/skipping, difficulty sleeping, and shortness of breath. The main symptoms observed in our study were similar to those observed in other studies, with differences in frequency and severity [19]. In patients with AF, these foremost symptoms may be linked not only to pathological features (i.e., impaired control of one’s heart rate and cardiac output, due to an irregular ventricular contraction and decreased cardiac output; sympathetic nerve inhibition; and loss of atrial function due to beta-blockers (the primary drugs used to treat AF)) [1] but also to an increased uncertainty due to physical discomfort [5]. The anxiety levels of patients in our study were lower than in previous studies [29]. This difference could be attributed to the high proportion of paroxysmal AF patients in our study, as well as the lower mean age of 61.55 years. The patients in our study had a moderate quality of life, with physical-related quality of life being lower than the mental-health-related quality of life. These findings are consistent with research [3,20,29].

Between the two cluster groups, which were formed based on the seven most common SCL symptoms, the cluster group with patients who complained of more symptoms had fewer patients living with families, and those patients reported less family support than the group with fewer symptoms. In a study that compared the severity of pain and fatigue in cancer patients who were divided into clusters of severe and mild symptoms, the group with severe symptoms was younger and often unmarried or living alone [14]. This is consistent with our findings, but the presence and support of family members may not directly reduce symptoms. However, it could be related to subjective health awareness, which provides psychological stability and ultimately reduces symptoms.

When clinical characteristics were compared, the group of patients who complained of the most symptoms had the most comorbidities. Furthermore, the cardiac output coefficient of this group was lower, and a higher proportion of patients in the group had higher NYHA classes. Furthermore, the duration of the disease, the number of hospital visits for symptoms, and the number of patients who underwent arrhythmia-related surgeries were all higher in this cluster.

These distinct differences in the clinical characteristics of the patients contradict previous research on symptom clusters. This disparity could be attributed to the severity of symptoms experienced by the patients in our study. Furthermore, in this study, patients with high severity and increased comorbidities complained of more symptoms. In the current study, the subjects had an average age of 61 years; and the number of comorbidities increased with age and severity of symptoms. As a result, a diverse set of symptoms was observed, contributing to an increase in frequency and severity.

These findings imply that symptom management is critical for patients with AF, particularly those with similar characteristics. In our study, cluster group 1, due to a higher frequency of symptoms, had higher levels of depression and anxiety than cluster group 2. The researchers discovered that group cluster variables had a significant impact on depression and anxiety. This finding is consistent with previous research, which found that anxiety and depression increased with the frequency and severity of symptoms [3,30]. Patients with AF generally have a lower quality of life than patients with other heart diseases, such as patients with heart failure or coronary artery disease [20]. The different factors that impact the quality of life include demographic factors, such as age, gender, occupation, cohabitation with family, and income [31]; clinical factors such as symptoms, NYHA class, and cardiac output coefficient; and psychological factors such as uncertainty, depression, and anxiety [17,30,32]. Existing research has also specifically shown that the relationship between symptoms and quality of life is significant in patients with AF.

In cluster group 1, physical and mental health-related quality of life was lower than in cluster group 2. Physical health-related quality of life was considerably lower than the mental health-related quality of life. Both cluster groups had low energy levels, implying that energy impacts the quality of life. As a result, we discovered that the overall level of energy and poor physical functioning were the primary factors that mitigated quality of life in patients with AF. Therefore, interventions to improve the physical health-related quality of life in patients with AF are required.

Regression analysis also revealed that symptom cluster group variables significantly impacted the quality of life. A daily AF-related frequent symptom lasting more than 2 h had a significant negative impact on quality of life [33]. Symptom control in patients with AF can have a significant impact on quality of life; thus, appropriate interventions are required. Furthermore, nursing interventions that can reduce the severity and frequency of multiple symptoms at once are required, rather than just relieving individual symptoms. Our findings suggest that certain strategies, such as targeting multiple-symptom interventions, can reduce depression and anxiety while improving AF patients’ quality of life. Symptom management also has the potential to reduce healthcare utilization, may have a significant impact on well-being, and contributes to clinical outcomes in AF patients.

## 7. Limitations

The first limitation of this study is that 62.3% of the patients included in the study sample had paroxysmal AF. Second, because the majority of the subjects were recruited from a tertiary outpatient hospital, the frequency and severity of symptom measurements may have been lower. Third, this study did not consider participants’ cardiovascular medication. Future studies should address these limitations by enrolling patients with more balanced AF diagnoses, as well as patients from multi-center different regions, medical locations, and periods. Participants’ medications should be surveyed, and their characteristics should be evaluated based on the type of cardiovascular medication they are taking. The timing of symptoms may influence their frequency and severity. It should be studied further.

## 8. Conclusions

The study’s analysis of survey data concentrated on the severity and frequency of symptoms in patients with AF. This resulted in the formation of two symptom clusters based on the seven most common symptoms observed in these patients. The cluster group with the most symptom complaints had less family support, increased disease severity, and a greater number of comorbidities. Depression and anxiety were also higher in this group, while the quality of life was low. Understanding the demographic and clinical characteristics of patients with AF with various symptoms aids in identifying and distinguishing vulnerable patients with the same disease, reduces anxiety and depression in patients, and improves patients’ quality of life. Management of the seven primary symptoms experienced by patients with AF could be another strategy for improving health-related outcomes. According to the findings of this study, nursing interventions that can reduce the severity and frequency of multiple symptoms simultaneously are critical. These targeted strategies can also help to reduce depression and anxiety in patients with AF while improving their quality of life.

## Figures and Tables

**Table 1:** Characteristics of participants (*N* = 175).

		Mean ± SD *n* (%)
Age		61.55 ± 11.50
Gender	Male	131 (74.9)
	Female	44 (25.1)
Education	Less than high school	50 (28.6)
	High school or above	125 (71.4)
Occupation	Yes	71 (40.6)
	No	104 (59.4)
Income	Low	93 (53.1)
	High	82 (46.9)
Living with family	Yes	143 (81.7)
	No	32 (18.3)
Currently drinks	Yes	92 (52.6)
	No	83 (47.4)
Currently smokes	Yes	56 (32.0)
	No	119 (68.0)
Classification of AF	Persistent AF (PeAF)	66 (37.7)
	Paroxysmal AF (PAF)	109 (62.3)
RFCA	Yes	108 (61.7)
Period since diagnosis		5.82 ± 7.83
Number of comorbidities		1.72 ± 1.85
Hypertension		78 (44.6)
Diabetes mellitus		38 (21.7)
Coronary artery disease		38 (21.7)
Heart failure		37 (21.1)
Arrhythmia (not AF)		36 (20.6)
LVEF		51.60 ± 7.83
NYHA	Class 1	88 (50.3)
	Class 2	70 (40.0)
	Class 3	17 (19.7)
Number of instances of health-care access (previous 6-month period)	once	109 (62.3)
	more	66 (37.7)

AF, atrial fibrillation; RFCA, Radiofrequency ablation; NYHA, New York Heart Association; LVEF, left ventricular ejection fraction.

**Table 2:** Frequency and severity of AF symptoms (*N* = 175).

	Frequency	Severity
	Mean ± SD *n* (%)	Mean ± SD *n* (%)
Heart racing	1.29 ± 1.18	1.03 ± 1.13
Fatigue	1.16 ± 1.15	1.01 ± 1.14
Dizziness/light headache	1.11 ± 1.09	1.01 ± 1.10
Tiredness/lack of energy	1.06 ± 1.10	0.85 ± 1.08
Heart fluttering/skipping	0.97 ± 1.19	0.73 ± 1.04
Difficulty sleeping	0.94 ± 1.30	0.82 ± 1.16
Shortness of breath	0.90 ± 1.12	0.73 ± 1.04
Hard to catch breath	0.55 ± 1.00	0.36 ± 0.78
Trouble concentrating	0.44 ± 0.81	0.35 ± 0.76
Chest pain when heart is racing	0.38 ± 0.84	0.26 ± 0.68
Sweating	0.33 ± 0.76	0.13 ± 0.51
Headache	0.28 ± 0.62	0.21 ± 0.52
Nausea	0.27 ± 0.72	0.14 ± 0.46
Poor appetite	0.27 ± 0.71	0.17 ± 0.48
Chest pain when heart is not racing	0.24 ± 0.61	0.18 ± 0.53
Feeling warm/flushed	0.18 ± 0.51	0.05 ± 0.24

Score range: Frequency 0–4 points; Severity 0–3 points.

**Table 3:** Participant AF symptoms, quality of life (QOL), and psychological distress (*N* = 175).

		Mean ± SD *n* (%)
Symptom Frequency		7.29 ± 6.45
Symptom Severity		7.43 ± 5.05
Anxiety		39.94 ± 9.94
Depression		13.10 ± 8.88
Quality of Life	Role limitation–physical	83.50 ± 23.65
	Physical function	82.31 ± 21.62
	Bodily pain	80.29 ± 13.95
	General health	61.09 ± 16.21
	Role limitation–emotional	81.90 ± 25.63
	Social function	75.50 ± 20.99
	Mental health	53.53 ± 1577
	Vitality	49.00 ± 15.03
	PCS	49.99 ± 10.67
	MCS	50.00 ± 8.80

PCS = physical component summary; MCS = mental component summary.

**Table 4:** AF Symptom of symptom cluster group (*N* = 175).

	Cluster Group 1 (*n* = 121 69.14%)	Cluster Group 2 (*n* = 54 30.86%)	*p* Value
	Mean ± SD *n* (%)	Mean ± SD *n* (%)	
Heart racing	1.69 ± 1.15	0.41 ± 0.68	<0.0001
Fatigue	1.57 ± 1.13	0.24 ± 0.47	<0.0001
Dizziness/light headache	1.40 ± 1.14	0.48 ± 0.63	<0.0001
Tiredness/lack of energy	1.41 ± 1.10	0.28 ± 0.56	<0.0001
Difficulty sleeping	1.33 ± 1.38	0.06 ± 0.30	<0.0001
Heart fluttering/skipping	1.17 ± 1.25	0.50 ± 0.92	<0.0001
Hard to catch breath	1.15 ± 1.20	0.35 ± 0.61	<0.0001

**Table 5:** Characteristics of symptom cluster groups (*N* = 175).

		Cluster Group 1 (*n* = 121)	Cluster Group 2 (*n* = 54)	*p* Value
		Mean ± SD *n* (%)	Mean ± SD *n* (%)	
Age		61.41 ± 11.83	61.85 ± 10.82	0.816
Gender	Male	90 (74.4)	41 (75.9)	0.829
	Female	31 (25.6)	13 (24.1)	
Education	Less than high school	35 (28.9)	15 (27.8)	0.877
	High school or above	86 (71.1)	39 (72.2)	
Occupation	Yes	48 (39.7)	23 (42.6)	0.718
	No	72 (60.3)	31 (57.4)	
Income	Low	62 (51.2)	31 (57.4)	0.453
	High	59 (48.8)	23 (42.6)	
Living with family	Yes	93 (76.9)	50 (92.6)	0.03
	No	28 (23.1)	4 (7.4)	
Currently drinks	Yes	57 (47.1)	26 (48.1)	0.899
	No	64 (52.9)	28 (51.9)	
Currently smokes	Yes	38 (31.4)	18 (33.3)	0.877
	No	83 (68.6)	36 (66.7)	
Classification of AF	Persistent AF (PeAF)	50 (41.3)	16 (29.6)	0.09
	Paroxysmal AF (PAF)	71 (58.7)	38 (70.4)	
RFCA	Yes	84 (69.4)	24 (44.4)	0.009
Period since diagnosis		6.04 ± 4.97	5.31 ± 5.25	0.03
Number of comorbidities		2.00 ± 2.01	1.44 ± 1.70	0.048
LVEF		51.56 ± 8.16	52.19 ± 7.02	0.023
NYHA	Class 1	55 (45.5)	33 (61.1)	0.016
	Class 2	51 (42.1)	19 (35.2)	
	Class 3	15 (12.4)	2 (3.7)	
Number of instances of health-care access (previous 6-month period)	Once	65 (53.7)	44 (81.5)	<0.0001
	More	56 (46.3)	10 (18.5)	
Anxiety		43.05 ± 9.66	32.96 ± 6.45	<0.0001
Depression		15.67 ± 9.00	7.33 ± 5.16	<0.0001
QOL	Role limitation–physical	78.62 ± 25.52	94.44 ± 13.61	<0.0001
	Physical function	77.81 ± 22.66	92.41 ± 14.91	<0.0001
	Bodily pain	77.36 ± 15.04	86.85 ± 7.96	<0.0001
	General health	56.32 ± 15.40	71.76 ± 12.55	<0.0001
	Role limitation–emotional	75.34 ± 27.52	96.60 ± 11.15	<0.0001
	Social function	68.80 ± 20.29	90.51 ± 13.50	<0.0001
	Mental health	48.76 ± 15.19	63.26 ± 12.09	<0.0001
	Vitality	44.92 ± 14.41	58.15 ± 12.18	<0.0001
	PCS	47.34 ± 10.88	55.93 ± 7.34	<0.0001
	MCS	48.84 ± 9.74	52.60 ± 5.43	=0.009

AF, atrial fibrillation; NYHA, New York Heart Association; LVEF, left ventricular ejection fraction; PCS, physical component summary; MCS, mental component summary.

**Table 6:** Relationships among quality of life, psychological distress, and symptom cluster group.

		R^2^	B	df	F	*p*	Covariate
Depression	Overall	0.255		6,618	3.162	<0.0001	Smoking, LVEF, NYHA class, number of instances of health-care access, number of comorbidities
	Symptom cluster		−8.110			<0.0001	
Anxiety	Overall	0.290		7,167	4.041	<0.0001	Drinking, LVEF, NYHA class, number of instances of health-care access, number of comorbidities.
	Symptom cluster		−9.859			<0.0001	
PCS	Overall	0.497		10,164	23.55	<0.0001	Anxiety, depression, smoking, drinking, LVEF, NYHA class, number of instances of health-care access, number of comorbidities
	Symptom cluster		6.741			0.001	
MCS	Overall	0.222		9,163	6.80	<0.0001	Anxiety, depression, smoking, drinking, RFCA, age, living with family
	Symptom cluster		4.626			0.002	

NYHA, New York Heart Association; LVEF, left ventricular ejection fraction; PCS, physical component summary; MCS, mental component summary.

## Data Availability

Data are available on request from the authors.
